# Osteotomies: Indications, Imaging Appearance, Surgical Techniques, and Complications

**DOI:** 10.3390/diagnostics15101184

**Published:** 2025-05-08

**Authors:** Arash Azhideh, Nastaran Hosseini, Sara Haseli, Chankue Park, Nitin Venugopal, Ali Abadi, Zahra Masroori, Eric Chen, Zachary Miller, David Camacho, Majid Chalian

**Affiliations:** Division of Musculoskeletal Imaging and Intervention, Department of Radiology, University of Washington, Seattle, WA 98105, USA

**Keywords:** osteotomy, postoperative complication, diagnostic imaging, magnetic resonance imaging, computed tomography, X-ray

## Abstract

Osteotomies can be performed on almost every bone and are commonly encountered indications for imaging studies. These procedures are employed to correct congenital, degenerative, and traumatic osseous deformities, ultimately improving both function and cosmetic appearance. It is crucial for radiologists to be aware of the wide range of surgical osteotomies and to be familiar with reporting clinically relevant imaging findings during surgical planning and post-operative follow-up. In this review, we discuss the indications, techniques, post-operative imaging appearance, and key reporting elements of commonly performed osteotomies, supported by comprehensive illustrative cases.

## 1. Introduction

Osteotomies are commonly performed surgical procedures that involve the deliberate cutting and realignment of bones. The success of these procedures largely depends on thorough pre-operative planning. Imaging plays a key role in both surgical planning and post-operative follow-up. Understanding the indications, surgical techniques, and expected imaging findings of various osteotomies is essential for accurate interpretation of radiographic studies. Radiologists must be expert at recognizing both normal and abnormal post-operative changes in osteotomy sites across various imaging modalities. This comprehensive review aims to explore osteotomies from head to toe, providing valuable insights for practicing radiologists.

### 1.1. Anatomic and Mechanical Axes in Deformity Correction

**Figure 1:**
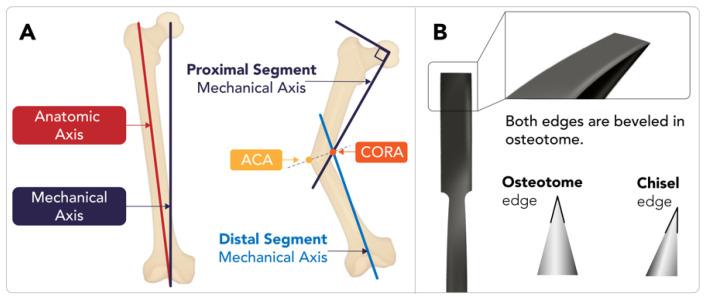
Osteotomy tools and basics of axes in deformity correction. In the schematic image (**A**), the anatomic axis, mechanical axis, center of rotation and angulation (CORA), and angle of correction (ACA) are illustrated. The CORA is defined as the intersection point of the proximal and distal mechanical axes of a deformed bone. The ACA is the angle required to correct the deformity [6]. (**B**) An osteotome is a surgical instrument resembling a chisel, but it has two beveled edges, unlike the single edge of a standard chisel. Osteotomes are widely used in orthopedic surgery, plastic surgery, and dental implantation for cutting or preparing bone, and they are available in various lengths and tip diameters.

From the surgeon’s perspective, the focus of osteotomy in deformity correction varies depending on the clinical context: It may involve redistributing load to relieve stress on a compromised joint, as in proximal tibial osteotomy, in order to delay degenerative joint disease. Alternatively, it may include creating new structural support to enhance joint stability, such as in acetabular osteotomies in patients with developmental hip dysplasia. In the long bones of the lower extremity, in particular, the necessary degree of correction can be estimated by analyzing the axes of alignment and rotation on radiographs. The basic principles of the (1) anatomic axis, (2) mechanical axis, and (3) center of rotation and angulation (CORA) can be helpful in these determinations. The anatomic axis is an imaginary line that passes through the center of a bone, representing the natural alignment of that bone in isolation. In a normal bone, this axis is parallel to diaphysis. A deformed bone, however, may have more than one anatomic axis. The mechanical axis is another imaginary line that represents the weight-bearing direction of a bone, normally representing a line that connects the center points of all the joints in a limb. The CORA in a deformed bone refers to the point around which the deformity should be rotated to achieve correction. The CORA is the intersection of one or more anatomic or mechanical axes and may lie outside the bone itself. The CORA can be used to determine the angulation correction axis (ACA), which is the line that bisects the angle formed by two axes intersecting at the CORA (Figure 1A). An osteotomy performed along the ACA that intersects with the CORA can be used to correct a deformity [1]. There are many more complex variations for determining the axes and CORA in cases of multiplanar and translated deformities, which are beyond the scope of this review.

Osteotomies are commonly performed to correct fracture malunion or non-union, aiming to restore proper bone alignment and/or healing. Delayed union is defined as the absence of normal healing progression radiographically or clinically 4 to 6 months after injury [2]. In cases of delayed union, where the bone ends fail to heal efficiently, fibrous tissue may develop along the free edges of the fracture, slowing down the formation of bridging bone. Surgical debridement along the fracture margins may provide fresh bone surfaces, facilitating fracture healing. Non-union is defined as failure of a fractured bone to heal or show progression toward union within the expected timeframe, usually 9–12 months [2]. Malunion occurs when a fractured bone heals an abnormal alignment and/or angulation, leading to functional impairment, pain, deformity, and limitations in joint movement. It frequently occurs in patients with reduced capacity for bony remodeling, such as the elderly, diabetics with poor glycemic control, immunosuppressed patients, smokers, malnourished individuals, and those with vascular disease, chronic kidney disease, or osteoporosis [3,4]. To correct malunion, an osteotomy is performed along the plane of the original fracture, typically intersecting with the CORA. The bone segments are then realigned into the desired position and fixed with screws, plates, or other devices to maintain stability [3].

In addition to addressing non-union and malunions, osteotomies can also correct joint malalignments. For example, in cases of chronic anterior cruciate ligament injury (ACL) with varus malalignment, osteotomies can alter the sagittal plane by influencing the tibial slope, thereby reducing ACL strain and risk of rupture [5].

We will discuss various specialized osteotomies in this review; however, the general principle of correcting the mechanical and anatomic axis of a bone or joint can be broadly applied to any malunited or congenitally malformed bone. While these procedures are not individually named here, it is important to note that a thorough understanding of the CORA helps in pre-operative planning and enables the surgeon to effectively address bone deformities.

### 1.2. Basic Osteotomy Techniques, Tools, and Hardware

#### 1.2.1. Osteotomy Techniques: Opening-Wedge, Closing-Wedge, and Dome Osteotomies

Common osteotomy techniques include opening-wedge, closing-wedge, and dome osteotomies. Beyond the mentioned techniques, several other methods are also performed based on the specific clinical indications and anatomical considerations.

Opening-wedge osteotomy typically involves creating a gap in the bone by opening a wedge-shaped section. Bone grafts or spacers or simple locking plates could be required to maintain the correction and/or fixation. In contrast, in closing-wedge osteotomy, a wedge of bone is removed, and then the remaining bone ends are repositioned and brought together to close the gap, which offers the benefit of bone-on-bone healing and allows for early weight-bearing without requiring bone grafts.

Lastly, dome osteotomy is employed for correcting coronal or sagittal plane deformities, such as those in the distal tibia [7]. DO is a semicircular osteotomy with an arc centered on the CORA [8]. Unlike opening- or closing-wedge osteotomies, DO allows for significant correction without requiring bone grafts or shortening. The DO technique utilizes specialized instruments, such as using a plate as a compass, to define the center of rotation for osteotomy. This instrument guides the creation of a dome-shaped cut and facilitates adjustable corrections during surgery while maintaining bone-to-bone contact. Concludingly, DO provides improved stability and reliable early bone healing at the osteotomy site due to bone-to-bone contact.

#### 1.2.2. Osteotomes and Fixation Methods

An osteotome is a specialized tool used during osteotomy to divide bones. The prototypical osteotome features a chisel-shaped functional end (Figure 1B). Numerous variations of osteotomes are used for specialized indications.

Additionally, fixation methods are essential in osteosynthesis to stabilize fractured bones and restore pre-injury function. Over the past three decades, these techniques have evolved significantly, advancing from wire fixation to the use of locking plates, screws, and microplates. Fixation methods are categorized into two main types: (1) Internal fixation: This approach involves mechanical devices directly in contact with bone. These devices support the fracture internally, promoting healing while minimizing external support. (2) External fixation: In this method, metallic pins are screwed into the bone through the skin and are connected to an external frame, allowing for adjustments and monitoring without further need for open surgical procedures [3].

### 1.3. Radiographic Assessment and Monitoring

Imaging techniques, such as radiography or computed tomography, are essential for pre-operative assessment and identification of post-operative complications such as hardware failure, non-union, or malalignment. While magnetic resonance imaging is less commonly used, it can be valuable for the diagnosis of soft tissue complications, including neurovascular damage or infection. Depending on the surgeon’s preferences, follow-up radiographs are obtained to monitor healing at regular intervals such as 6 weeks, 12 weeks, 6 months, 12 months, and then annually [9]. It is important to note that the specific monitoring schedule may vary depending on the type of osteotomy and patient factors (e.g., smoking, diabetes, obesity, and non-compliance with weight-bearing protocols) [10].

**Figure 2:**
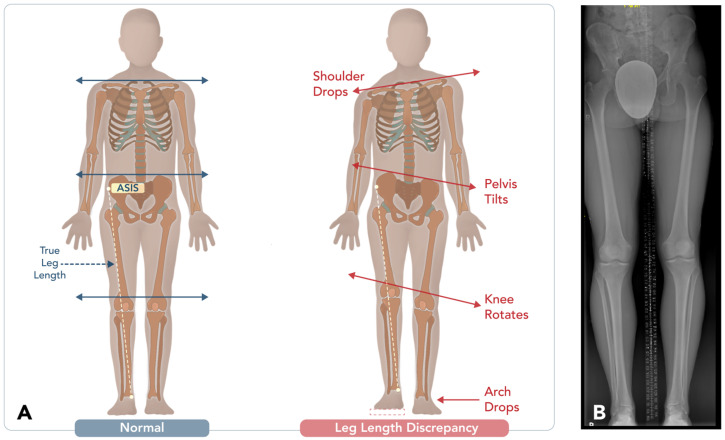
Assessment and consequences of leg length discrepancy (LLD). (**A**) Illustrative diagram comparing normal alignment with the compensatory biomechanical changes associated with leg length discrepancy. In the presence of LLD, compensations may include shoulder drop, pelvic tilt, knee rotation, and foot arch collapse, all contributing to altered gait and musculoskeletal pain. (**B**) Long-leg standing radiograph (scanogram) used to quantify the degree of LLD.

Accurate imaging is critical for pre-operative planning, but several challenges can arise when obtaining reliable images. Correct positioning, appropriate imaging modalities, and consideration of how deformities may affect biomechanical axis measurements are essential. Malalignment during imaging can lead to inaccurate measurements, ultimately affecting the surgical outcome. For example, in cases of limb length discrepancy (LLD) (Figure 2), the limb must be positioned in its natural anatomical alignment, ensuring that the CORA is correctly defined. Imaging must also reflect the full length and alignment of the limb to provide an accurate pre-operative assessment. The most commonly used imaging techniques include orthoroentgenograms, scanograms, and teleoroentgenograms.

In orthoroentgenograms, an extralong cassette is used with the patient laying still, and three sequential radiographic exposures centered over the hip, knee, and ankle are carried out. A teleoroentgenogram is carried out with the same cassette length but with the patient standing, and there is one single exposure centered over the knee joint. In a scanogram, the patient is lying next to a calibrated ruler, and three sequential exposures centered over the hip, knee, and ankle are carried out with a standard-sized cassette [11].

Alternatively, compensatory techniques such as adjusting the pelvic tilt or using blocks can help correct the alignment of the affected limb. Additionally, in patients with spinal deformities, it is crucial to take standing X-rays with proper posture to assess alignment of the spin under normal weight-bearing conditions. Imaging obtained while the patient is lying down or seated may fail to show the true alignment [12,13,14].

### 1.4. Post-Operative Complications

Monitoring fracture healing is a crucial part of post-op care. Post-operative complications, though uncommon, are classified into three clinical categories to guide management and determine the need for additional interventions: Class 1: complications requiring no further pharmacological or surgical intervention, Class 2: complications requiring additional or extended non-operative care, and Class 3: complications requiring additional or revision surgery or long-term medical management [15]. Criteria for assessing union may vary among orthopedic surgeons. Radiographs can be limited by their two-dimensional nature and metalwork interference. Callus formation usually appears within 6–7 weeks, with the cortical bridging being a key indicator of union, although it may take up to three months. If clinical and radiographic assessments during regular imaging evaluations suggest a delayed union or non-union, a CT scan using a metal artifact reduction algorithm can confirm the diagnosis and quantify the percentage of healing across the osteotomy site. CT scans are particularly effective at revealing osteotomy edges, bone graft integration, and early hardware failure, which may not be apparent on conventional radiographs. However, CT scans may sometimes over-diagnose non-union due to differing definitions of union. Additionally, while the radiation dose for peripheral limb CT is relatively low, it increases significantly for areas closer to radiosensitive tissue, such as the shoulder and hip, which requires careful consideration during imaging. MRI and nuclear imaging may be useful for detecting soft tissue complications, though their role in non-union prediction remains limited [16].

## 2. Osteotomy Approaches

Different regions of the body, including the skull and maxillofacial area, upper and lower extremities, hips, vertebrae, and ribs, require tailored osteotomy approaches depending on their specific anatomical and functional demands. In the following sections, we will explore the diverse range of osteotomy techniques employed across the mentioned anatomical regions to highlight indications, challenges, and outcomes.

### 2.1. Skull and Maxillofacial Osteotomies

#### 2.1.1. Craniotomy

Craniotomy typically involves the removal of a piece of the calvarium, referred to as the “bone flap”. After surgery, the bone flap is usually replaced and fixed with plates and screws. There are several craniotomy variations, including the burr hole (a small hole created with a surgical drill), craniectomy (the bone flap is not replaced after removal), or cranioplasty (the bone flap is replaced with either a bone graft or synthetic material) [17].

Craniotomy techniques involve scalp incision, followed by soft tissue dissection. One or more burr halos are created using a surgical drill. A saw is then used to connect these burs, allowing the surgeon to remove the bone flap and expose the dura mater. The dura is transected to provide access to the cranial cavity [18].

**Figure 3:**
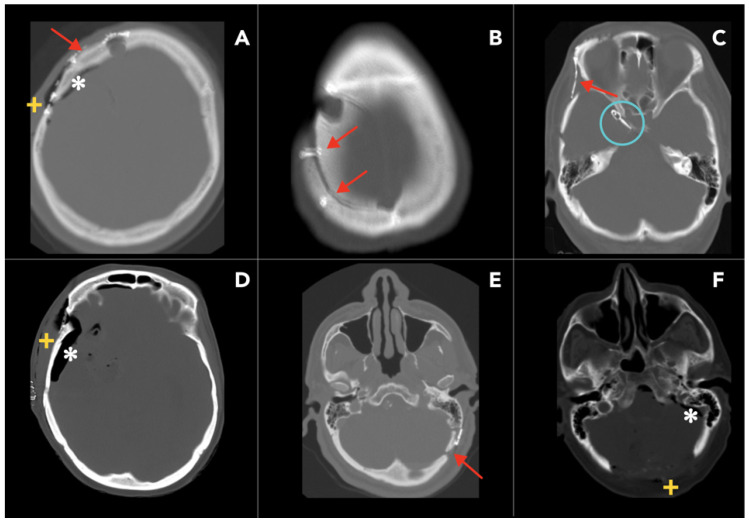
Post-operative CT imaging of various craniotomy approaches. The images illustrate various craniotomy procedures (red arrows) and their associated post-operative findings. (**A**) Frontal craniotomy with fixation hardware, pneumocephalus (*), and soft tissue swelling (+). (**B**) Parietal craniotomy. (**C**) Pterional craniotomy and an aneurysm clip in its proper location (blue circular outline). (**D**) Temporal craniotomy. (**E**) Retro-sigmoid craniotomy. (**F**) Sub-occipital craniotomy with typical post-surgical findings.

The specific approach to the craniotomy depends on the area of the cranial cavity that needs to be accessed. The type of craniotomy is named after the portion of the calvarium removed. For example, frontal or parietal craniotomy is performed to access the anterior skull base, frontal lobe, third ventricle, and sellar space (Figure 3A,B). Pterional (fronto-spheno-temporal) craniotomy is used to access the anterior circulation, cavernous sinus, frontal and temporal lobes, and supra-sellar space (Figure 3C). Temporal or sub-temporal craniotomies are used to access the temporal lobes and the middle cranial fossa (Figure 3D). Occipital and retro-sigmoid craniotomies are used to access the posterior fossa (Figure 3E,F) [18].

In post-operative evaluation, a normal post-operative pneumocephalus could be expected on early imaging (Figure 3), but this should be resolved within three weeks after surgery. Other complications of craniotomy include hematoma, infection (e.g., soft tissue infection, abscess, subdural empyema, and osteomyelitis), air embolism, dural sinus perforation, and, rarely, tension pneumocephalus (Figure 3).

#### 2.1.2. Lateral Nasal Osteotomy

Lateral nasal osteotomy (LNO) is performed as part of a rhinoplasty to modify the width or projection of the dorsal nasal bridge and base. Primarily, the reasons for LNO include improving the aesthetic appearance of the nose, but it can occasionally be performed for functional indications, such as addressing nasal airway obstruction [19].

**Figure 4:**
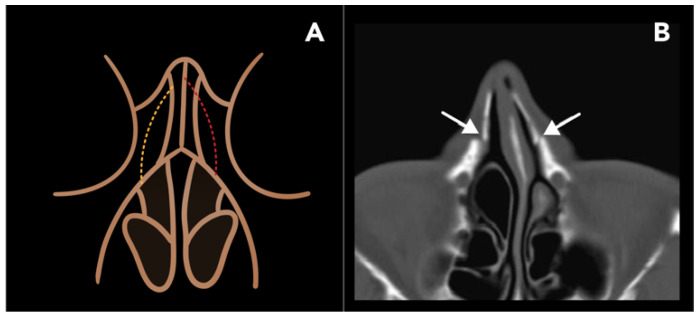
Lateral nasal osteotomy. (**A**) A coronal schematic image of the face at the level of the nasal and orbital bones demonstrates the low-to-low (yellow-dotted line) and low-to-high (red-dotted line) osteotomy lines. (**B**) Post-operative axial CT image shows defects in the bilateral nasal processes of the maxillae (arrows), with medial displacement of the lateral nasal walls.

In rhinoplasty, LNO is particularly used to close an open roof deformity of the nasal aperture following a humpectomy (resection of the dorsal nasal bridge). LNO techniques are designed in order to preserve the base of the nasal aperture, preventing collapse of the nasal bridge [20]. There are two main LNO approaches, categorized as follows: (1) low-to-high (LTH) and (2) low-to-low (LTL) (Figure 4A). Both techniques begin from a lateral point of the piriform aperture, anterior to the attachment of the inferior turbinate. The terms “low” and “high” refer to the perspective of the surgeon with the patient laying supine and the tip of the nose oriented towards the ceiling and can be thought of as posterior and anterior, respectively. In LTH, the osteotomy begins at the piriform aperture and travels caudally, terminating at the intercanthal line, ending higher on the dorsal nasal bridge. In LTL, osteotomy takes a more horizontal course and remains low to the level of the inner canthus. This approach liberates a larger portion of the lateral nasal wall, allowing for a greater degree of correction compared to the LTH technique. For this reason, the LTL approach is generally preferred, unless only a minor degree of correction is needed [19,20].

Post-operatively, CT scans may demonstrate defects in the bilateral nasal bones and should not be mistaken for a traumatic nasal bone fracture (Figure 4B). LNOs are typically fixed in place with radiolucent material such as sutures or adhesive tape, which are not visible on CT. While CT scans are not routinely obtained for post-operative follow-up, they may be carried out if complications, such as hemorrhage, occur or for other unrelated reasons. Other potential complications include periorbital edema, ecchymosis, breathing disturbance, and nasal collapse.

#### 2.1.3. Reduction Malarplasty

Reduction malarplasty (RM) is a cosmetic procedure used to reduce the size of prominent zygomas. It is among one of the most commonly performed facial-contouring surgeries in certain parts of the world. There are no specific pre-operative findings, and the determination of zygomatic prominence is typically subjective. A maxillofacial CT scan may be obtained for surgical planning [21].

**Figure 5:**
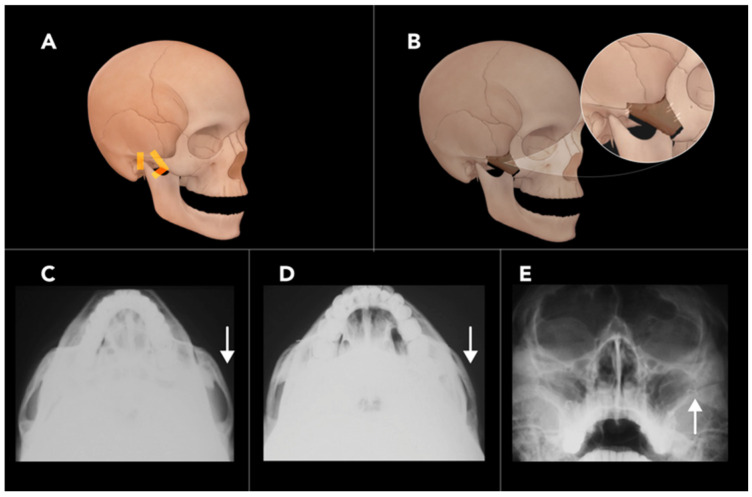
Reduction malarplasty. (**A**) An illustration of the face and skull demonstrates an L-shaped osteotomy of the zygomatic body and an additional osteotomy of the zygomatic arch, allowing for repositioning of the zygomatic fragment. (**B**) Post-operative illustration shows fixation hardware correctly placed. Axial radiographs (**C**,**D**) of the zygoma demonstrate reduced bowing of the zygomatic arch following surgery (arrow) (**D**). A Water’s view radiograph (**E**) highlights post-surgical changes (arrow).

The most common technique for RM combines trans-oral and percutaneous approaches to free the zygomaticomaxillary complex. A trans-oral osteotomy is L-shaped. The long arm of the L is parallel to the axis of the zygoma, extending from the lateral orbital rim medially into the lateral aspect of the maxilla, just lateral to the posterior wall of the maxillary sinus. The short arm runs perpendicularly to the long arm and exits the inferior cortex of the maxilla (Figure 5A,B). A percutaneous osteotomy involves a vertical cut or in-fracture through the posterior zygomatic arch, made through an incision that parallels the sideburn of the hair. The resulting free fragment resembles a zygomaticomaxillary complex fracture (tripod fracture) but leaves the inferior orbital rim intact. This fragment is then medially rotated and stabilized with plate and screw fixation [22].

Post-operative imaging of RM will demonstrate a medial rotation of the inferior aspect of the zygoma (Figure 5C,E). Complications of RM include non-union, restricted range of motion, and facial nerve injury. Cheek drooping is another post-operative complication, which can result from non-union or inadequate fixation. If the osteotomy is not adequately stabilized, the masseter muscle, which attaches superiorly to the zygomatic bone, may cause the zygoma to sag or break, leading to cheek drooping [22,23].

#### 2.1.4. Bilateral Sagittal Split Osteotomy

Bilateral sagittal split osteotomy (BSSO) is used to correct a variety of mandibular deformities, including horizontal mandibular excess, deficiency, or asymmetry. It is also performed to reposition the mandible anteriorly or posteriorly in cases of retrognathism or prognathism, respectively. In patients requiring more than 10–12 mm of mandibular set-back, intra-oral vertical ramus osteotomy may be considered an alternative option to BSSO for patients [23].

**Figure 6:**
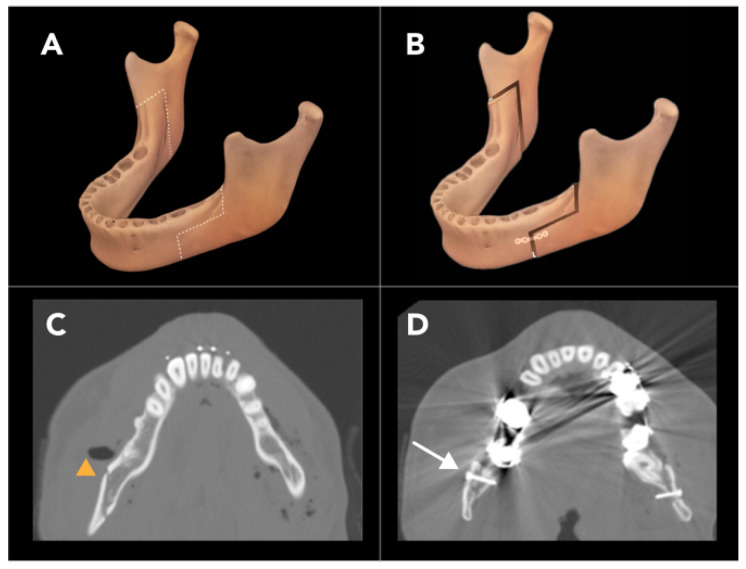
Bilateral sagittal split osteotomy (BSSO). Illustrations of the mandible depict the pre-operative osteotomy line (**A**) and the post-operative results following BSSO (**B**). Axial reconstructed CT images post-BSSO show the osteotomy site of the right-mandibular body (yellow arrowhead (**C**)) and screw fixations (arrow (**D**)).

BSSO is typically performed using a trans-oral approach. There are various techniques for the osteotomy cuts used for BSSO, but all the variations generally involve making cuts at the mandibular body and ramus on both sides of the mandible, creating a free-floating segment of the anterior mandible that can be repositioned as necessary. A critical consideration during BSSO is maintaining the integrity of the mandibular foramen, which contains the inferior alveolar nerve and artery. To avoid damaging the mandibular foramen contents, a series of partial-thickness cuts, often referred to as a “lingual split”, are used to work around the foramen. This lingual split is the defining feature of BSSO, giving the procedure its name [24] (Figure 6A,B). Accordingly, pre-operative imaging is essential to estimate the location of the mandibular foramen relative to the cortices of the mandible. Conventional helical CT has been shown to be superior to panoramic radiographs and cone-beam CT in locating the mandibular canal pre-operatively [25].

There are various BSSO modifications, such as the Dal Pont modification and the Hunsuck modification, with the latter technique widely used for BSSO. In both techniques, a horizontal osteotomy (lingual osteotomy, LO) is performed through the mandibular ramus, and a vertical osteotomy (buccal osteotomy, BO) is made through the mandibular body. The LO is a partial-thickness cut that is made from the inner cortex of the mandible, extending to the lateral border of the mandibular foramen. Likewise, the BO is a partial-thickness cut made from the outer cortex, also terminating laterally to the mandibular foramen. A vertical or oblique cut is then made to join the LO and BO, resulting in a Z-shaped or zig-zag cut (Figure 6A,D). This frees the body of the mandible from the rami, allowing for repositioning. The segment is then fixed in place with plates and screws. The Hunsuck modification, which has been associated with better outcomes than the Dal Pont technique, involves terminating the lingual osteotomy just past the lingula of the mandible instead of extending it through the posterior cortex of the mandibular ramus [26].

Injury to the inferior alveolar nerve is a significant concern during BSSO, with direct nerve transection reported in up to 3.5% of cases and long-term neurologic deficit reported in 10–30% of patients. Other complications associated with BSSO include unanticipated fractures of the condylar neck, buccal plate, or lingual plate, as well as injury to the inferior alveolar artery, avascular necrosis, and condylar resorption. Worsening temporomandibular joint pain can also occur following BSSO.

### 2.2. Upper Extremity Osteotomies

#### 2.2.1. External Rotation Humeral Osteotomy

External rotation humeral osteotomy (ERO), also known as de-rotational osteotomy, is used to correct excessive internal rotation of the humerus. The most common cause of humerus internal rotation deformity is brachial plexus injury, often due to obstetric brachial plexus palsy (e.g., Erb’s palsy), particularly affecting the C5-6 nerve roots. This injury results in weakness of shoulder external rotation and internal rotation contracture [27]. The deformity is also accompanied by a degree of the forearm and elbow flexion contractures, and patients often have difficulty reaching their face with the affected arm. These combined deformities significantly impair upper extremity function, making comprehensive imaging of the shoulder, elbow, and forearm essential for surgical planning [28]. The goal of the ERO procedure is to improve external rotation and thereby increase arm abduction and external rotation. ERO is often performed in older pediatric patients but should ideally be planned before severe stiffness develops [29]. The choice of pre-operative imaging modality depends on the patient’s age and the specific anatomical considerations. Ultrasound and MRI are useful for evaluation of obstetric brachial plexus palsy in patients younger than 5, since the joint composition in this age group is cartilaginous. MRI is particularly helpful in evaluating the morphology of the glenoid according to the Birch classification (concave–flat, convex, or biconcave) [30,31]. In older patients with internal rotation deformity, pre-operative radiographs may demonstrate findings of glenohumeral dysplasia, characterized by humeral head flattening, glenoid retroversion, and posterior subluxation of the humeral head due to persistent muscular imbalance [32].

**Figure 7:**
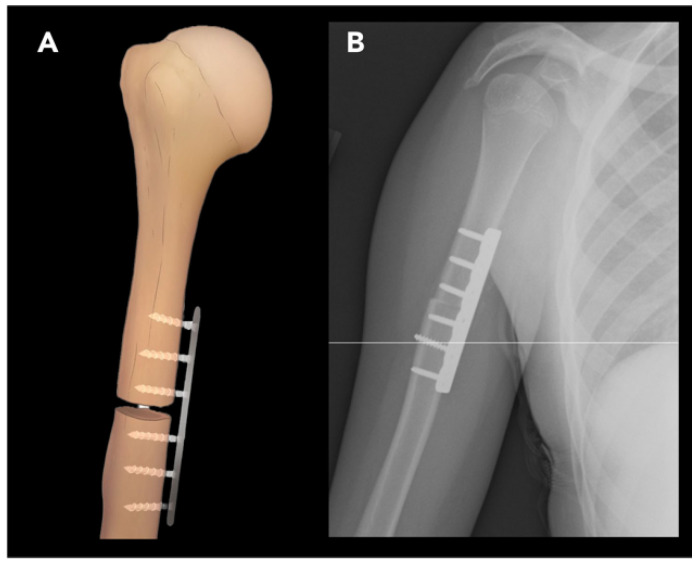
External rotation humeral osteotomy. (**A**) Illustration of the humerus shows a transverse osteotomy performed proximal to the insertion of the deltoid muscle; fixation hardware is medially placed, and the distal humerus is externally rotated, depending on the severity of the deformity. (**B**) Frontal radiograph demonstrates plate and screw fixation, indicating appropriate alignment of the osteotomy fragments.

Surgical technique involves a transverse osteotomy in the middle third of the humerus, just proximal to the deltoid tuberosity, to improve the alignment of the deltoid. The distal fragment is then externally rotated and fixed in place using a compression plate and screws (Figure 7) [33].

Post-operatively, some degree of increased posterior subluxation of the humeral head is expected and is not an indication for further intervention. As a consequence of ERO, some degree of internal rotation is also sacrificed, which may impair the patient’s ability to reach their abdomen with the ipsilateral arm [33].

#### 2.2.2. Ulnar Shortening Osteotomy

Ulnar shortening osteotomy is performed to address various conditions such as ulnocarpal abutment syndrome, ulnar styloid impingement syndrome, and Madelung deformity (congenital dyschondrosis of the distal radial physis), all of which cause continuous or intermittent chronic excessive loading across the ulnocarpal join.

When performing pre-operative radiological assessment for ulnar shortening osteotomy, it is essential to consider several key factors. These include the measurement of ulnar variance on the AP view, referring to the relative length of the distal ulna compared to the distal radius at the wrist joint [34]. Additional factors include classification of the distal radioulnar joint (DRUJ) type and the lunate bone morphology (flat, round or triangular). The presence of DRUJ osteoarthritis is critical in determining the surgical approach. Additionally, it is important to recognize radiographic findings that may suggest an underlying structural abnormality on the ulnar side of the wrist. These findings may include lunate subsidence, positive ulnar variance, and palmar carpal displacement [35].

**Figure 8:**
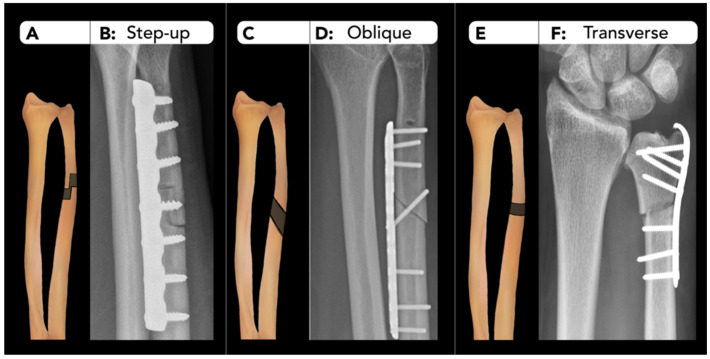
Ulnar shortening osteotomy. (**A**–**F**) Various techniques of ulnar shortening osteotomy. (**A**,**B**) The step-cut approach, which involves an interlocking step pattern at the osteotomy site, resulting in a larger contact surface area and enhanced rotational stability. (**C**,**D**) The oblique approach, characterized by an angled cut across the ulna, allowing for gradual compression and control shortening. (**E**,**F**) The transverse approach, featuring a horizontal cut perpendicular to the ulna’s long axis, which simplifies the surgical procedure and provides straightforward alignment during fixation. Corresponding radiographs with fixation hardware for each technique are also shown.

Ulnar shortening osteotomy can be performed with step-cut, oblique, or transverse approaches (Figure 8). Outcome is generally similar across these techniques.

#### 2.2.3. Distal Radius Corrective Osteotomy

Distal radius corrective osteotomy is performed to correct deformities or malalignment of the radius or distal radioulnar joint. The primary goal of surgery is to restore the proper alignment of the radiocarpal articulation and distal radioulnar joint. Malunion is a frequent source of pain following distal radial fractures and is a common indication for this procedure. Malunion is a complication of distal radial fractures [36] and can accelerate arthrosis at the distal radioulnar joint, disrupt biomechanics of wrist flexion, and impair forearm rotation, leading to pain and weakness [37]. Ulnar shortening osteotomy may occasionally be performed address this condition but is less commonly used.

Pre-operative planning for distal radius corrective osteotomy typically relies on radiographs in the anteroposterior and lateral views to evaluate volar or dorsal angulation, radial inclination, ulnar variance, and radial length [38]. There are no defined radiographic criteria for distal radial fracture malunion, but radial inclination < 10°, volar tilt > 20°, and positive ulnar variance > 2 mm are considered indicators of possible clinically significant malunion [37]. Radiographs of the contralateral wrist are often obtained as part of the pre-operative workup as a reference point for a patient’s normal wrist alignment [36]. While these two-dimensional images provide valuable information, they may not fully capture complex deformities, particularly rotational abnormalities. Recent studies have highlighted three-dimensional imaging techniques, such as CT scans, for more precise evaluation. In addition, the use of 3D patient-specific guides has been introduced to effectively enhance pre-operative planning. PSGs assist in accurately positioning the osteotomy and fixation hardware, improving the alignment post-operatively [38].

**Figure 9:**
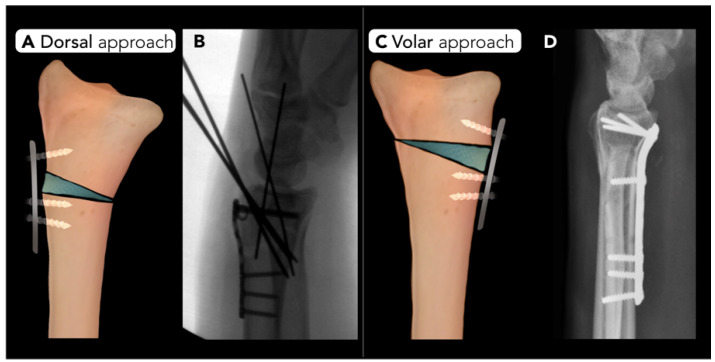
Distal radial corrective osteotomy. Two surgical approaches for correcting distal radius deformities. (**A**) Dorsal approach: An opening-wedge corrective osteotomy with plate and screw fixation to correct dorsal tilt. (**B**) Intraoperative lateral radiograph of the wrist shows the dorsal corrective osteotomy stabilized with a plate, screw, and K-wire. (**C**) Volar approach: An opening-wedge corrective osteotomy with plate and screw fixation is utilized to correct volar tilt. (**D**) Post-operative radiograph highlights the distal radial corrective osteotomy with plate and screw fixation addressing post-traumatic deformity.

Distal radius corrective osteotomy can be performed using a dorsal or volar approach. Typically, a transverse osteotomy is created in the anteroposterior plane, followed by correction of volar tilt and radial inclination. Plate and screw fixation is used to secure the distal fragment in a near-anatomic position. The wedge-shaped gap created by the correction is filled with cancellous bone graft to enhance healing (Figure 9) [39].

Radial osteotomy complication rates can vary depending on the technique. The most common dorsal fixation complications include extensor tendon irritation or rupture, necessitating hardware removal in the majority of patients. Complications of volar-sided surgery include metalwork failure, positional loss, and ulnar shortening. Additionally, median neuropathy remains a concern, and locking T-plates can sometimes cause radial deviation of the epiphysis, increasing nerve compression risk [40]. Relative contraindications for distal radial corrective osteotomy include radiocarpal or intercarpal osteoarthritis and osteoporosis [36].

#### 2.2.4. Scaphoid Corrective Osteotomy

Scaphoid corrective osteotomy is used to manage symptomatic scaphoid non-union or malunion. The scaphoid is the most commonly fractured carpal bone, with up to 10% of scaphoid fractures progressing to non-union due to its tenuous blood supply.

Pre-operative imaging often reveals a non-united or malunited scaphoid waist fracture with or without a humpback deformity (volar tilt of the distal fragment of the scaphoid). The lateral intra-scaphoid angle, measured as the angle between two lines perpendicular to the proximal and distal articular surfaces of the scaphoid, respectively, is typically increased to greater than 30° in cases associated with humpback deformity [41]. In cases of scaphoid non-union, pre-operative MRI is performed to assess the vascularity of the proximal pole, which may assist in surgical planning; however, intra-operative assessment remains the best method for evaluating proximal fragment vascularity in scaphoid non-union [42,43].

**Figure 10:**
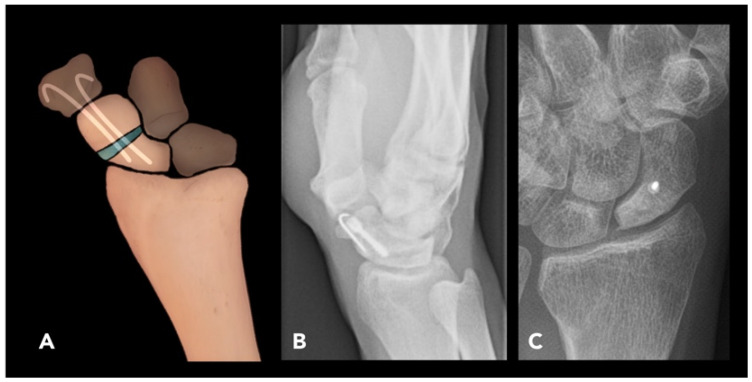
Scaphoid corrective osteotomy. (**A**) Illustration demonstrates a scaphoid corrective osteotomy with bone graft (blue) placed at the osteotomy site. The graft is stabilized by 2–3 K-wires, which are typically removed 12–16 weeks post-operatively. While a fixation screw can be used, it is not the preferred method. (**B**,**C**) Post-operative radiographs: Lateral radiograph shows osteotomy and fixation with a screw and K-wire for scaphoid fracture (**C**). Scaphoid view radiograph demonstrates near-complete fusion without complications in the scaphoid following the corrective osteotomy with screw fixation.

The technique involves performing a single transverse osteotomy at the scaphoid waist or at the site of non-union. The osteotomy is filled with bone graft and stabilized with 2–3 K-wires, often supported with a transfixing screw for additional stability. The wires are typically removed 12–16 weeks post-operatively (Figure 10).

Complications of scaphoid corrective osteotomy include avascular necrosis and repeat non-union or malunion, which can be detected on post-operative radiographs. Radiographs may reveal increased radiodensity in the proximal segment compared to the capitate bone. CT scans can provide detailed images in identifying areas of increased radiodensity. MRI is particularly valuable; on T1-weighted sequences, osteonecrosis is defined by decreased signal intensity in the proximal segment. This signal reduction can be further assessed by comparing it to the muscle signal within the same image [44,45].

To minimize hardware-related issues, slightly shorter screws than the specified length should be used, and central screw placement should be verified intra-operatively with fluoroscopy. Non-vascularized, vascularized, or arthroscopic-assisted bone grafting can be used to treat non-union with or without AVN, providing similar outcomes. Complications of malunion, such as dorsal segmental intercalated instability (DISI) and scapho-navicular advanced collapse, are also commonly encountered [46]. Humpback deformity and DISI can be managed with anterior cortico-cancellous wedge grafting or temporary K-wire fixation combined with cancellous bone grafting [47]. The presence of peri-scaphoid arthritis on pre-operative radiographs may be a contraindication to corrective osteotomy.

### 2.3. Lower Extremity Osteotomies

#### 2.3.1. High Tibial Osteotomy

High tibial osteotomy (HTO) is performed to treat angular deformities of the knee to prevent uni-compartmental knee arthritis, most commonly affecting the medial compartment. The primary indication for HTO is varus deformity on weight-bearing radiographs. The osteotomy serves to re-distribute axial load, alleviate pain, and slow the progression of medial compartment degeneration [10]. The HTO procedure is typically considered as a bridge to knee arthroplasty and does not replace the role of joint replacement.

Pre-operative evaluations include weight-bearing AP views radiographs of both knees in full extension. Bilateral weight-bearing posteroanterior tunnel views in 30° of flexion can be used in conjugation with the AP views to increase the radiographic detection of OA [48,49]. Some authors suggest obtaining additional weight-bearing images from the hip to ankle to visualize the Mikulicz line, drawn from the center of the femoral head to the center of the ankle joint, to estimate the mechanical axis of the lower extremity [10,50]. This line is typically positioned 4 ± 2 mm medial to the center of the knee. A deviation from this range indicates a valgus or varus alignment if the line shifts laterally or medially, respectively [51]. In certain conditions, such as patient height, obesity, or poor image quality, the femoral head may not be clearly visible. In these cases, the tibiofemoral angle can be assessed by calculating the anatomical femoral axis and assumption of an anatomic–mechanical femoral angle of 6°. The difference between this assumption and the measured value is considered as the degree of deformity. If the ankle joint is indistinct, the tibial axis line should be drawn from the center of the knee to a midpoint on the visible end of the tibial shaft [51]. Another key consideration in pre-operative imaging is the posterior tibial slope, measured as the angle between the tibial joint surface plane and a perpendicular tangent to the long axis of the tibia. This influences sagittal plane alignment and is important for orthopedic surgeons, as ligamentous laxity or deficiencies must be accounted for in estimating necessary slope correction. This parameter can be assessed on lateral knee radiographs. In certain clinical settings, MRI of the knee may also be useful to rule out further causes of medial knee pain, including medial meniscal deficiency, anterior cruciate ligament or posterior cruciate ligament deficiency, or osteochondral defects.

Ideal candidates for HTO are those under the age of 65 with isolated medial osteoarthritis, good range of motion, and no ligamentous instability. It is estimated that HTO can delay the need for total knee arthroplasty by 8–10 years, and thus, for older patients, it may be preferred to go directly to uni-compartmental or total knee arthroplasty [52]. HTO contraindications include severe medial compartment damage, tri-compartmental or patellofemoral OA, concurrent infection, BMI > 40, age > 65, tobacco use, and limited range of motion [53]. Advantages of HTO include preservation of native knee anatomy, less function restriction, and the possibility of delaying arthroplasty [54]. However, HTO also has drawbacks, such as prolonged healing time, the risk of non-union, potential inadequate pain relief, and increased complexity of future arthroplasty [55,56].

**Figure 11:**
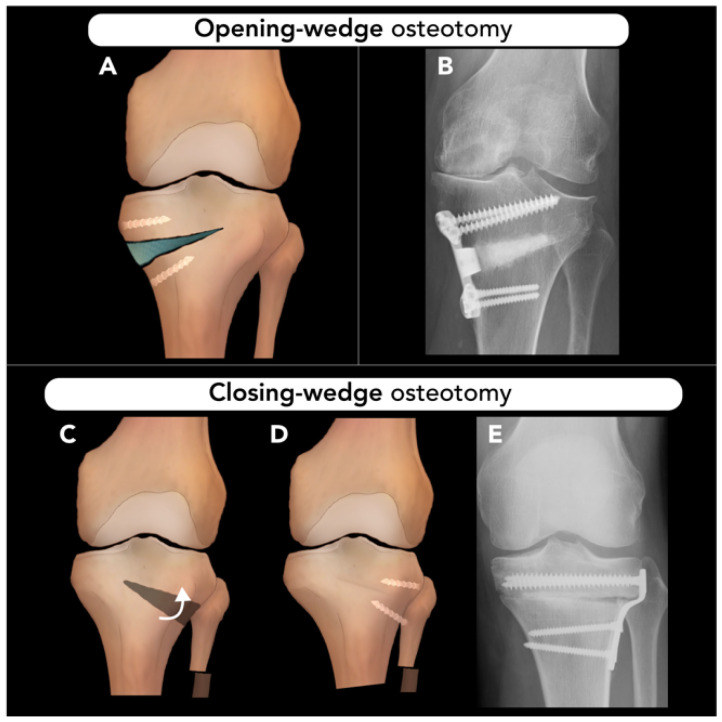
High tibial osteotomy. Two techniques for high tibial osteotomy. (**A**,**B**) Opening-wedge osteotomy: opening-wedge osteotomy of the medial proximal tibia, where the osteotomy site is filled with graft material and stabilized with plates and screws. (**C**–**E**) Closing-wedge osteotomy: A wedge-shaped osteotomy is created along the lateral tibial metaphysis, and a proximal fibular osteotomy is shown. The distal tibia segment is rotated to close the osteotomy (curved arrow (**C**)) eliminating the resulting wedge (**D**), and the cut sections are then apposed and stabilized with hardware (**E**).

HTO can be performed on a varus knee using the medial opening-wedge, lateral closing-wedge, or dome osteotomy techniques [57]. The medial opening-wedge technique involves performing a wedge osteotomy from the medial tibial metaphysis and filling the defect with bone graft, followed by plate and screw fixation around the defect (Figure 10A,B) [50]. A lateral closing-wedge approach was historically used, which involves resecting a wedge from the lateral tibial metaphysis and proximal fibula. However, this approach can cause disruption of the proximal tibiofibular joint and, most importantly, increase the difficulty of subsequent knee arthroplasty (Figure 11C–E) [10].

In valgus knees, a medial closing-wedge HTO can be carried out. Alternatively, a lateral opening- or medial closing-wedge osteotomy in the supracondylar region of the distal femur, called a distal femoral osteotomy, can also be considered, especially if the valgus is femoral-based, the patellofemoral joint is involved, and there is patellar maltracking [58].

Both opening-wedge and closing-wedge osteotomies are widely used in HTO for the treatment of medial or lateral compartment osteoarthritis. These techniques are becoming more popular due to advances in biological joint reconstruction rather than reverting to arthroplasty. Additionally, while the opening-wedge HTO technique is precise and preserves the proximal tibiofibular joint, it may cause additional tension on the medial collateral ligament [59,60]. Closing-wedge HTO also presents challenges such as the increased technical difficulty of two tibia cuts, possible peroneal nerve injury, an increase in posterior tibial slope, and complications in performing future knee arthroplasty [59,60]. According to a meta-analysis by Wu et al., opening-wedge HTO leads to a greater range of motion, increased posterior slope, and a lower patellar height compared to closing-wedge HTO, with no significant differences in knee pain, functional scores (e.g., Lysholm knee scoring scale, Hospital for Special Surgery Knee-Rating Scale), surgery time, and duration of hospitalization [61].

Post-operative standing hip-to-ankle radiographs are obtained annually to monitor alignment and detect any loss of correction (e.g., changes in the mechanical axis or the medial proximal tibial angle) in addition to clinical evaluation. Ensuring a neutral foot position in both pre-operative and post-operative imaging is essential [10], as foot internal rotation reduces the apparent varus deformity and mechanical axis deviation, while external rotation increases both. One of the most significant post-operative complications of HTO is lateral cortical hinge fracture, which most commonly occurs when less than 1 cm of lateral cortex is left intact when performing a medial OWO [62]. Recurrence of varus alignment is also a common and expected chronic complication of HTO. The medial proximal tibial angle, which is the angle between the articular surface of the tibial plateau and the tibial shaft, should be measured on post-operative radiographs to identify recurrent malalignment.

#### 2.3.2. Talar Neck Osteotomy

Talar neck osteotomy (TNO) is performed to correct malunited talar neck fractures. Malunion of talar neck fracture occurs in up to 10% of cases, most commonly due to inadequate primary reduction in and fixation of displaced talar neck fractures. Malunion shortens the medial column of the hindfoot, leading to forefoot varus, impaired biomechanics of the Chopart joint complex (a joint between the midfoot and hindfoot), restricted sub-talar joint motion, and an inability to evert the foot [63]. Uncorrected talar neck malunion results in a high incidence of post-traumatic subtalar arthritis (16% to 100%) and AVN of the talus (up to 38%). However, occurrence of AVN may be more closely linked to an emergent reduction in displaced fractures rather than to surgical fixation timing [64,65].

Radiographic assessment of malunited talar fractures should include weight-bearing radiographs of both the affected and contralateral foot and ankle. CT scan is also recommended to assess the degree of sub-talar and tibiotalar joint incongruity. In patients with symptomatic sub-talar arthritis, joint fusion may be preferred over TNO. If AVN is suspected and involves more than one-third of the talar body, which is likely a contraindication for TNO, MRI is the preferred imaging modality. In this circumstance, necrectomy with fusion is required instead [66].

**Figure 12:**
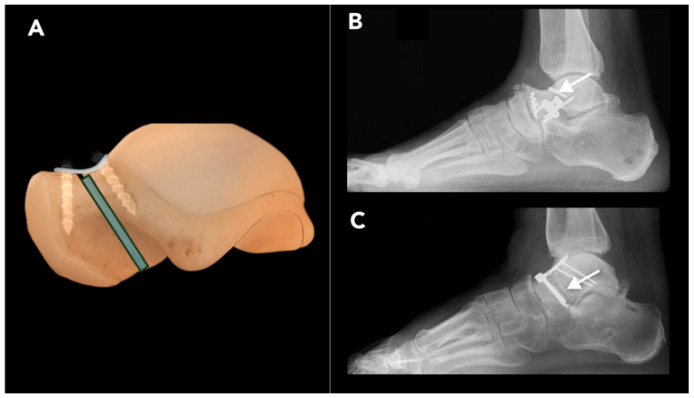
Talar neck osteotomy. Post-operative talar neck osteotomy: (**A**) Illustration depicting the post-operative appearance of the talus following a talar neck osteotomy. The talus fragments are rotated to achieve the desired correction. A graft (blue rectangle) is placed in the osteotomy site and stabilized with hardware fixation. (**B**,**C**) Post-operative radiographs from patients who underwent this procedure showing visible fracture planes (arrow) and the presence of buttress plates and screws used for fixation.

The medical approach under fluoroscopy is typically preferred for TNO. Intra-operative inspection of the sub-talar and talonavicular articular cartilage may reveal advanced arthritis not evident on imaging, in which case corrective fusion or arthroplasty should be performed instead of osteotomy. If the surgeon decides to proceed with joint-preserving TNO, an osteotomy is performed along the former fracture plane, and the varus deformity is corrected under fluoroscopy with the aid of K-wires. The resulting defect is then filled with bone graft and transfixed with small fragment screws or plate (Figure 12) [67].

Complications such as non-union or AVN may be seen on post-operative imaging and may require corrective fusion. Anterior impingement may occur during dorsiflexion if plate fixation is performed, although this will not be evident on post-operative images. Post-traumatic arthritis is the most prevalent consequence after talar neck fractures, with the sub-talar joint being the most frequently affected, followed by the tibiotalar and talonavicular joints, respectively [68].

#### 2.3.3. Calcaneal Osteotomy

Calcaneal osteotomy (CO) is performed to correct various deformities of the hindfoot, most commonly pes plano-valgus and cavo-varus. These deformities often involve structural abnormalities in more than one plane, and the surgical techniques reflect the multiplanar nature of the these conditions [69]. Common techniques for calcaneal osteotomy include single-plane translational osteotomy, lateral column lengthening (Evans) osteotomy, closing-wedge (Dwyer) osteotomy, and rotational-type osteotomy (Z-osteotomy) [70,71]. These techniques may be used in combination to achieve near-anatomic alignment of the hindfoot. While conservative measures (e.g., orthotics) are often attempted as first-line therapy, surgery is often required for definitive correction [71].

**Figure 13:**
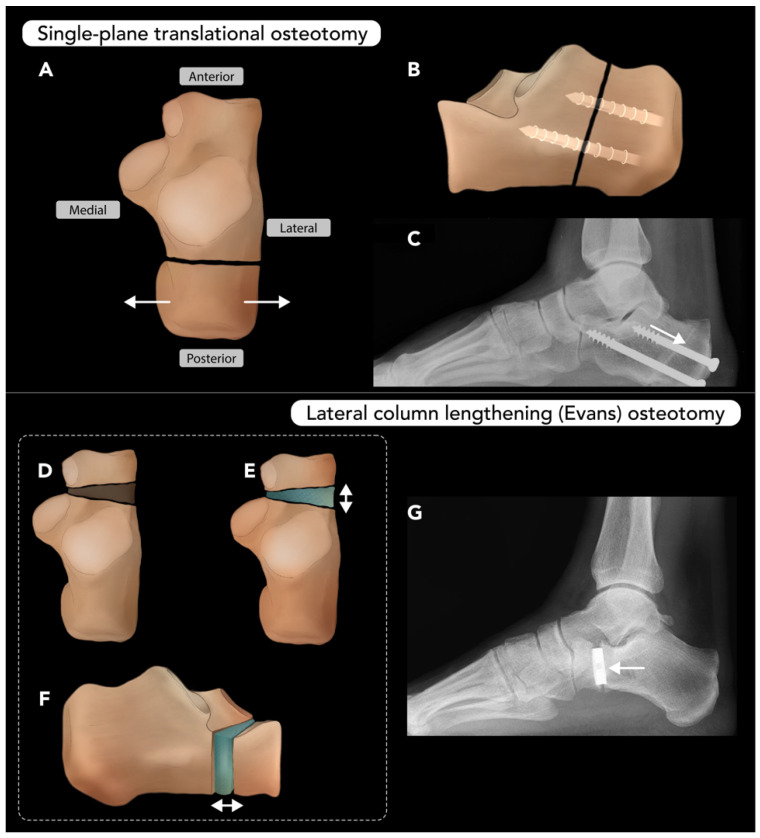
Calcaneal osteotomy. Two types of calcaneal osteotomies. (**A**–**C**) Single-plane translational osteotomies: Illustration (**A**) from a superior view showing medial or lateral repositioning of the free calcaneal fragment (arrow) to correct plano-varus or cavo-varus deformities. Illustration (**B**) showing a lateral view of the post-operative calcaneus with screws transfixing the osteotomy site. Lateral ankle radiograph of a patient who underwent this procedure, highlighting the oblique osteotomy plane (arrow) and screw fixation (**C**). (**D**–**F**) Lateral column lengthening (Evans) osteotomy: Illustrations showing the osteotomy cut through the neck of the calcaneus (**D**), lateral column lengthening (double arrow (**E**,**F**)), and graft insertion within the wedge (blue (**E**,**F**)). Lateral ankle radiograph of a patient post-Evans osteotomy, with a transverse osteotomy and graft placement at the osteotomy site (arrow (**G**)).

The translational osteotomy technique involves a single transverse cut through the body of the calcaneus with subsequent translation of the calcaneal tuberosity (Figure 13A–C). For plano-valgus deformity, the tuberosity is translated medially, while for cavo-varus deformity, the tuberosity is translated laterally. The lateral column lengthening (Evans) osteotomy technique involves a cut through the neck of calcaneus, followed by insertion of a bone graft wedge to lengthen the lateral column. This can be performed with or without a medial translational osteotomy or plantar flexion osteotomy of the medial cuneiform bone (Figure 13D–G). Closing-wedge (Dwyer) osteotomy is a variation of the translational osteotomy used to correct cavo-varus deformity, involving additional resection of a lateral wedge of bone to redistribute axial forces toward the lateral heel. Complex rotational-type, or Z-shaped, osteotomy allows for three-dimensional manipulation of the calcaneal tuberosity, which is then stabilized with fixation [71].

The primary contraindication to calcaneal osteotomy that radiologists must be aware of is sub-talar joint osteoarthritis. In such cases, osteotomy is unlikely to resolve symptoms, and sub-talar arthrodesis is preferred over CO [70]. Complications are rare but can include over- or under-correction, tarsal tunnel syndrome, sural nerve damage, and non-union or malunion.

Radiographic assessment of calcaneal osteotomies focuses on alignment, healing, and the status of graft material. Weight-bearing radiographs, such as the hindfoot alignment view (Saltzman), are used to define post-operative alignment [72]. Calcaneal osteotomies rarely result in non-union or malunion. According to Greenfield et al., this occurs most commonly in patients with systemic comorbidities, such as vitamin D deficiency. Patients at risk may benefit from osteo-inductive methods, such as bone morphogenetic protein at the osteotomy site, to improve post-operative outcomes [73].

#### 2.3.4. Hallux Valgus Osteotomy

Hallux valgus (HV), or bunion deformity, is characterized by a medial deviation of the first metatarsal and lateral deviation of the hallux. It typically presents as a visible deformity of the great toe and is often associated with pain around the first metatarsophalangeal joint due to bursitis, arthritis, or entrapment of the medial dorsal cutaneous nerve of the foot. Symptoms are often exacerbated by wearing tight shoes [74]. Hallux valgus osteotomy, or first metatarsal osteotomy, is commonly performed to correct HV deformity.

**Figure 14:**
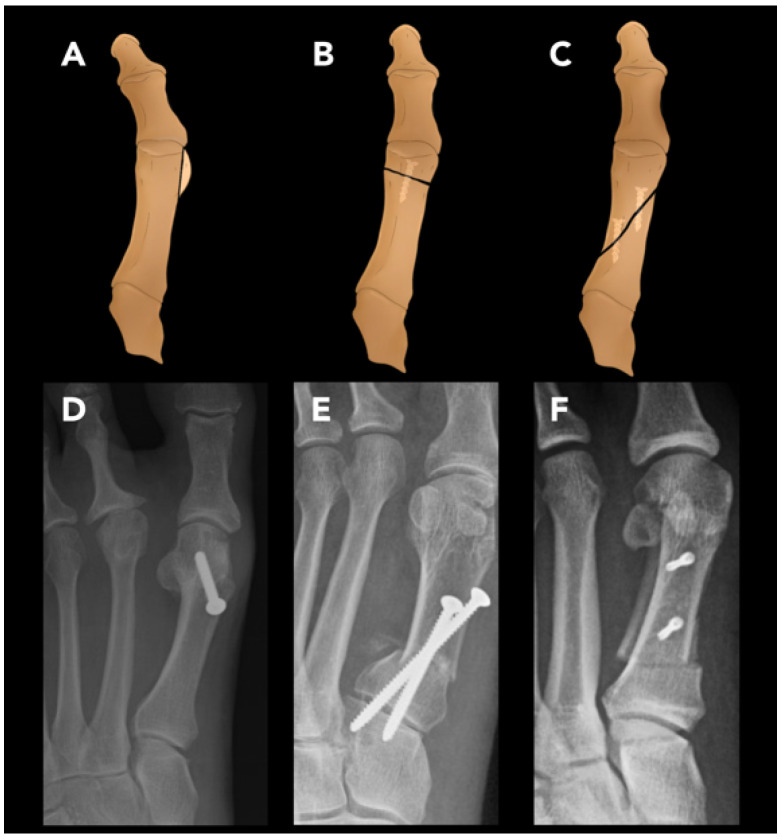
Hallux valgus osteotomy. Various techniques of first metatarsal osteotomy for hallux valgus correction. (**A**) Coronal view of the first metatarsal bone, illustrating the medial metatarsal head being “shaved” prior to performing osteotomies. (**B**) Distal osteotomy of the first metatarsal head. (**C**) Proximal metatarsal osteotomy of the same bone. (**D**) Post-operative radiographic findings following distal hallux valgus osteotomy, with the hallux valgus angle measuring 10° and the intermetatarsal angle measuring 7°, both within normal ranges. (**E**,**F**) Proximal metatarsal osteotomy, specifically the proximal chevron cut (**E**) and the scarf cut (**F**).

The primary radiographic findings of hallux valgus are an elevated hallux valgus angle (HVA) and intermetatarsal angle (IMA). The HVA is the angle formed by the axis of the proximal phalanx of the great toe and the first metatarsal. A normal HVA is less than 15° (Figure 13A). An HVA exceeding 15°, 20°, and 40° are criteria for mild, moderate, and severe HV, respectively. The IMA is the angle between the axis of the first metatarsal and second metatarsal. A normal IMA is less than 9° (Figure 14A). Values exceeding 9°, 11°, and 18° are criteria for mild, moderate, and severe HV, respectively. Additional considerations for surgical planning include the position of the hallux sesamoids and the congruence of the first MTP joint, which are also assessed on foot radiographs [75].

Determining the choice of technique is guided by the severity of HVA and IMA. For symptomatic patients with mild HV, distal osteotomy is performed, which involves making a cut through the first metatarsal head and shifting the distal fragment laterally (Figure 14B,D). A common variation of distal HVO, sometimes referred to as a chevron osteotomy, is characterized by a V shape at the first metatarsal head. For moderate HV, a proximal osteotomy is preferred and involves a cut at the metatarsal base (Figure 14C). Both distal and proximal HVO can be carried out with a chevron or scarf (Z-osteotomy) cut (Figure 14B,C). For severe HV, a double metatarsal osteotomy, combining proximal and distal techniques, is often preferred. Adjunct procedures performed along with HVO include the following: An Akin osteotomy, which is a medial CWO at the base of the proximal first phalanx to address markedly elevated IMA. A bunionectomy is another common adjunct procedure that reduces the deformity by direct resection of the medial eminence of the first metatarsal condyle [70].

Radiographs are the gold standard for post-operative assessments. Weight-bearing X-rays in the AP, lateral, and oblique views are crucial for evaluating deformity correction, bone consolidation, and overall foot function. It is recommended to perform X-rays on the day of surgery and again at six weeks post-operatively. Lateral and oblique views are particularly useful for assessing the first metatarsal, tarsometatarsal, and first MTP joints [76].

### 2.4. Pelvic Osteotomy

Pelvic osteotomy is performed to correct abnormal acetabulum morphology, such as that in developmental dysplasia of the hip (DDH). DDH is characterized by an abnormally shallow acetabulum, increasing the risk of hip subluxation and placing additional stress on the acetabular rim, leading to labral and acetabular articular cartilage injury [77]. Labral tears are present in up to 90% of patients with symptomatic hip dysplasia and are a common presenting symptom. If left uncorrected, these conditions almost universally lead to early hip osteoarthritis, making timely diagnosis and treatment critical. Pelvic osteotomy aims to re-orient the acetabulum by moving to the joint center more medially and provide adequate acetabular coverage of the femoral head [78].

Ultrasound is the primary imaging tool for evaluating DDH, using Graf staging for classification. Conventional radiography becomes the primary imaging modality after 4–6 months for pre-operative planning, including AP and lateral projection of the hip. Key measurements include the acetabular index (AI) and lateral center-edge angle (LCA) parameters, with a prominent finding in DDH being a reduced LCA [79]. The LCA, measured on AP radiographs of the pelvis, is the angle between a vertical line through the center of the femoral head and a second line drawn through the center of the femoral head extending to the most lateral aspect of the acetabular roof (Wiberg method) or sourcil (Ogata method). An LCA of 25–40° is considered normal, while <20° is diagnostic of hip dysplasia [80]. Upturning of the acetabular sourcil is another characteristic finding of DDH.

There are generally three types of pelvic osteotomies: (1) re-directional osteotomies (e.g., triple innominate, peri-acetabular osteotomy, and spherical osteotomies), (2) reshaping osteotomy (e.g., Pemberton, Dega, and San Diego osteotomies), and (3) salvage procedures (e.g., Chiari, Shelf osteotomies).

The triple innominate technique involves osteotomies of the ilium, ischium, and pubis to isolate and realign the acetabulum.

**Figure 15:**
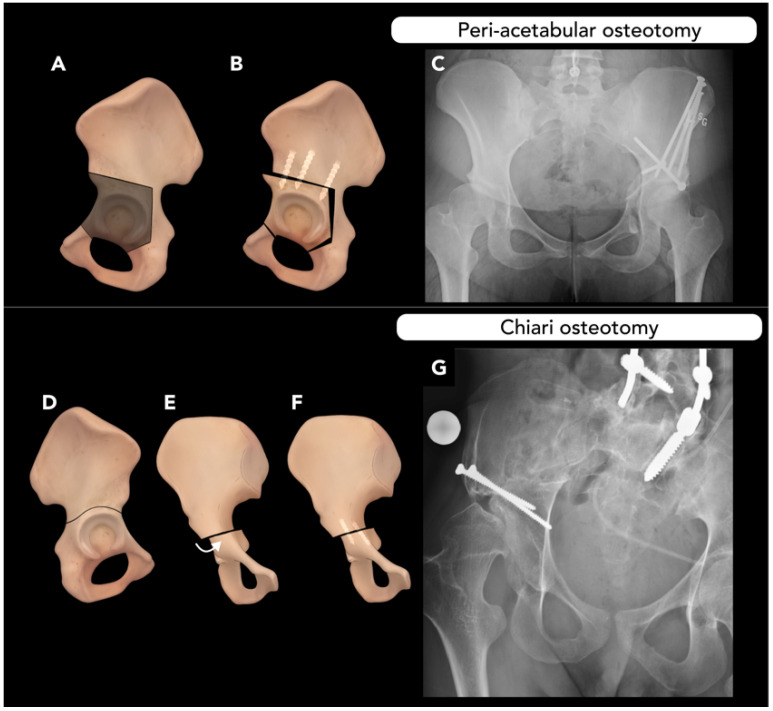
Pelvic osteotomy. Two surgical techniques for correcting acetabular dysplasia. (**A**–**C**) Peri-acetabular osteotomy: Illustrations demonstrating peri-acetabular osteotomy line (**A**), with a K-wire used to re-position the bone into the desired position. The post-operative appearance is depicted with three screws transfixing the ileum to the free bone fragment (**B**). Post-operative pelvic radiograph showing the osteotomy with multiple screw fixations (**C**). (**D**–**G**) Chiari osteotomy: Illustrations depicting the Chiari osteotomy line (**D**), where the acetabulum is displaced medially, resulting in the iliac component being positioned more laterally to provide increased coverage for the femoral head (Curved arrow, **E**). Following adequate acetabular coverage, screws are used transfix the osteotomy (**F**). A post-operative pelvic radiograph of a patient who underwent Chiari osteotomy is shown (**G**).

Periacetabular osteotomy (PAO), such as the Bernese or Ganz techniques, is a modification of the triple innominate technique, with cuts closer to the margins of the acetabulum. The PAO approach maintains the integrity of the acetabulum posterior column, requires less fixation, and provides a greater degree of freedom for acetabulum re-orientation (Figure 15A–C) [81]. Both the triple innominate osteotomy and the PAO methods mobilize the quadrilateral plate, allowing for medialization of the acetabulum if necessary. PAO has shown superior long-term outcomes compared to the triple innominate osteotomy method [82].

Spherical osteotomy, another type of re-directional osteotomy, can also be performed with a circumferential cut even closer to the margins of the acetabulum but violates the joint capsule, making it a less favorable option for DDH correction [83].

For skeletally immature patients, re-shaping osteotomies targeting the tri-radiate cartilage, such as Pemberton or Dega osteotomies, are other alternative surgical options for the correction of dysplasia [81].

Chiari osteotomy, a medial displacement pelvic osteotomy (Figure 15D–G), is usually reserved as a salvage procedure in cases of inadequate femoral head coverage. It has become less common since the 1990s due to advances in other pelvic osteotomies and total hip arthroplasty [84].

The most common complication following PAO is non-union, particularly in patients with high BMI. Over-correction can lead to subsequent femoral head over-coverage, presenting similarly to pincer-type femoroacetabular impingement. Heterotopic ossification may also occur and, if particularly severe, can impair hip mobility. Injury to the lateral femoral cutaneous nerve is another potential complication [81].

Post-operative imaging in pelvic osteotomy is crucial to assess the consolidation of osteotomy sites and correct positioning of the surgical implants. It is essential to evaluate whether the Y-shaped growth plate has been injured and to identify any foreign material within the joint spaces. Common post-operative complications include osteonecrosis of the femoral head, implant failure, pseudoarthrosis, defined as a lack of bone healing for more than six months, and recurrent malposition. In cases of pseudoarthrosis following triple osteotomy, it is important to recognize that this condition is often asymptomatic at the pubis and ischium. Therefore, routine radiographic follow-up is crucial for early detection and intervention, ensuring optimal surgical outcomes [85].

In addition to pelvic osteotomies, some patient may require femoral de-rotational osteotomy to address deformities, such as excessive femoral anteversion, which is characterized by an abnormal angular difference between the femoral neck axis and the trans-condylar axis of the knee. Normal femoral anteversion is typically 10–15° in adults [86]. Several clinical manifestations, such as internally rotated gait, patella–femoral instability, and anterior knee pain, might result from excessive femoral anteversion [87]. Patients with clinical symptoms and radiological findings suggestive of severe anteversion of the femur are candidates for femoral de-rotational osteotomy. Modalities such as MRI, ultrasound, CT, and specialized radiographs (e.g., the Dunn/Rippstein view) are used to assess femoral version, with MRI and CT considered the gold standards. The Dunn/Rippstein view is an AP view in which the patient lies supine, with hips and knees flexed at 90°, and with legs abducted 20° [88].

De-rotational osteotomy techniques vary based on the location of the deformity, as well as the expertise of the surgeon: (1) at the proximal femur (corrected with intertrochanteric osteotomies, secured using angled blade plates), (2) diaphyseal (corrected with an intra-medullary rod), or (3) distal femur (corrected with supracondylar osteotomies and secured with locking plates).

Intertrochanteric osteotomy is frequently used for patients with hip dysplasia or femoral rotational abnormalities. This technique, performed at the proximal femur, allows for the correction of not only rotational deformities but also varus/valgus and flexion/extension abnormalities. While acetabulum re-orientation has emerged as the standard treatment for hip dysplasia, femoral de-rotational and inter-trochanteric osteotomies remain important options for addressing severe rotational deformities. This highlights the complementary nature of these procedure and their significance in achieving comprehensive alignment and symptom relief that cannot be fully addressed by acetabular correction alone [89].

### 2.5. Vertebral and Rib Osteotomies

#### 2.5.1. Thoracolumbar Vertebral Osteotomy

Vertebral osteotomy (VO) is performed to correct spinal deformities and restore normal spinal curvature. This procedure can be performed for most cases of symptomatic sagittal or coronal imbalances, including scoliosis, kyphosis, or degenerative changes. This spinal malalignment can result in pain, neurologic symptoms, and cosmetic deformities. Sagittal spinopelvic alignment has shown the strongest overall association with the degree of pain and disability, making it the primary focus of VO procedures [90].

The Schwab classification system is widely used to categorize VO techniques into six grades based on the extent of resection. Grade 1 osteotomies involve removing the inferior facet and joint capsule, while grade 2 osteotomies add resection of the ligamentum flavum, inferior and superior articulations, as well as additional posterior spinal components such as lamina or spinous processes. As with grade 1, grade 2 osteotomies necessitate antecedent anterior column movement. Grade 3 osteotomies involve partial wedge excision of the posterior vertebral body and pedicles. A portion of the vertebral body and discs above and below the osteotomy remain. Grade 4 osteotomies require broader wedge resections of the vertebral body, including the posterior components and pedicles, as well as the removal of an endplate and a portion of a neighboring disc. Grade 5 osteotomies involve complete vertebral level removal, including adjacent discs and ribs from the thoracic region constitutes, while grade 6 osteotomies extend beyond grade 5 ones in their scope of resection [90].

Whole-spine erect radiographs are the cornerstone of pre-operative evaluation for spinal imbalance and planning the corrective procedures. Radiologists must be familiar with a number of different key spinopelvic measurements, such as lumbar lordosis/pelvic incidence mismatch (LL-PI), pelvic tilt (PT), and sagittal vertical axis (SVA), used to evaluate alignments. Among these, pelvic incidence (PI) is unique, as it is the only constant morphologic parameter, regardless of patient position. All the other positional parameters can only be measured accurately with the matin standing, using a full lateral spinal X-ray. The SVA is determined using the C7 plumb, drawn vertically from the center of the C7 vertebral body, parallel to the vertical axis of the image. Ideally, the C7 plumb line should intersect the posterosuperior corner of S1. A deviation greater that 2 cm anterior or posterior to the posterosuperior corner of S1 is considered a positive or negative sagittal balance, respectively [91].

There are numerous VO variations that can provide different degrees of correction, tailored to certain imbalances. VO variations, in escalating order of the degree of correction they provide, include (1) Smith–Peterson osteotomy, (2) pedicle subtraction osteotomy, (3) bone–disc–bone osteotomy, and (4) vertebral column resection [92].

**Figure 16:**
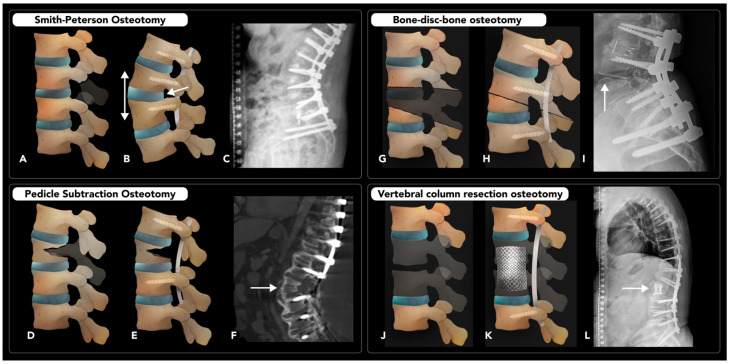
Thoracolumbar vertebral osteotomy. Various spinal osteotomy techniques. (**A**–**C**) Smith–Peterson osteotomy (SPO): Illustrations depicting the resection of posterior elements and subsequent screw fixation (**A**,**B**). This procedure achieves spinal lordosis by shortening the posterior column (arrow (**B**)) and lengthening the anterior column (double-headed arrow (**B**)). Post-operative lateral spinal radiograph of a patient who underwent SPO at the L2 level (**C**). (**D**–**F**) Pedicle subtraction osteotomy (PSO): Illustrations showing a wedge resection of the vertebral body, with shortening of the middle column without lengthening of the anterior column (**D**,**E**). Post-operative sagittal CT reconstructed image of a patient who underwent PSO at the L2 level (arrow (**F**)). (**G**–**I**) Bone–disc–bone osteotomy (BDBO): Illustrations showing the resection of the intervertebral disc with adjacent endplates (**G**), and subsequent bone-to-bone apposition and fixation (**H**). Post-operative lateral spinal radiograph of a patient who underwent BDBO the L3-4 level (arrow (**I**)). (**J**–**L**) Vertebral column resection osteotomy (VCR): Illustrations depicting the resection of multiple levels of whole vertebra segments, including the disc, with replacement using a metal cage (**J**,**K**). Post-operative lateral spinal radiograph of a patient who underwent VCR at the L1-2 level with a cage insertion (arrow (**L**)).

Smith–Peterson osteotomy (SPO) is a closing-wedge VO used to correct a positive sagittal balance caused by straightening of the normal thoracic kyphosis (e.g., flexion deformity or “flat back syndrome”). SPO involves resecting the lamina, facet joints, and ligamentum flavum at one or more levels, resulting in posterior column shortening and extension of the spine (Figure 16A–C). It can provide 5–10 corrections at each level and is typically indicated for milder flexion deformities with 6–8 cm of positive sagittal balance, such as Scheuermann’s disease. In SPO, a defect is created in the posterior column only, leaving the spine stabilized by the intact anterior and middle columns. Concludingly, the non-fused intervertebral disc, acting as a hinge for wedge closure, is stabilized using posterior instrumentation [93].

Pedicle subtraction osteotomy (PSO) is another closing-wedge VO that can provide a larger level of correction than SPO, with up to 30 corrections per level. PSO is generally indicated for more severe flexion deformities, such as those with 10–12 cm of positive sagittal balance. PSO involves the resection of both pedicles and part of the vertebral body at a particular level, leaving a segment of the anterior cortex as a hinge (Figure 16D–F). Unlike SPO, PSO affects both the posterior and middle columns, allowing it to correct deformities in fused segments [93,94].

Disc–bone osteotomy (BDBO) is a subset of VO used to correct even more severe imbalances than previously mentioned PSO and SPO techniques, particularly an imbalance centered around a disc space. This technique provides up to 60° of correction and involves resecting a disc and two adjacent endplates (type I BDBO) (Figure 16G–I). BDBO variations may involve a closing-wedge VO at the level above (type II BDBO) or at the levels above and below (type III BDBO) the resected disc. Due to the significant degree of instability created, stabilization requires fixation at three levels above and two levels below the osteotomy [93].

Vertebral column resection (VCR) offers the most extensive degree of correction of the techniques discussed here, with up to 70° of sagittal imbalance correction. VCR involves resection of one or more vertebral segments and replacement with a metal cage or structural autograft. A long-segment arthrodesis is then performed around the level(s) of the osteotomy to ensure stability (Figure 16J–L). This technique may also include costotransversectomy if the thoracic spinal vertebra is removed. VCR is also used for conditions like resection of a tumor centered in a vertebra [94].

Post-operative plain radiographs are useful for assessing hardware integrity and spinal alignment. Standard projections may include AP, lateral, and weight-bearing views, with additional extension and lateral bending views. CT is also used for evaluating fusion status and complications such as pseudoarthrosis or infection. However, CT exposes patients to significant ionizing radiation, which could be a concern if there is a repeated need for scans. MRI, on the other hand, is the imaging of choice for assessing soft tissue complications such as inflammation, fibrosis, or herniation. Both modalities could be affected by artifacts from metal implants, which can be mitigated with using specific hardware material and advanced artifact reduction techniques [95]. Complications rates vary, depending on the technique used for VO and the extent of correction. Common complications include nerve root impingement, epidural bleeding, coronal decompensation, and pseudoarthrosis. More aggressive corrections, such as PSO and VCR, carry higher risks compared to SPO. Neurological complications occur in approximately 2% of SPO cases, 9% of PSO cases, and up to 14% of VCR cases [96].

#### 2.5.2. Rib Osteotomy

**Figure 17:**
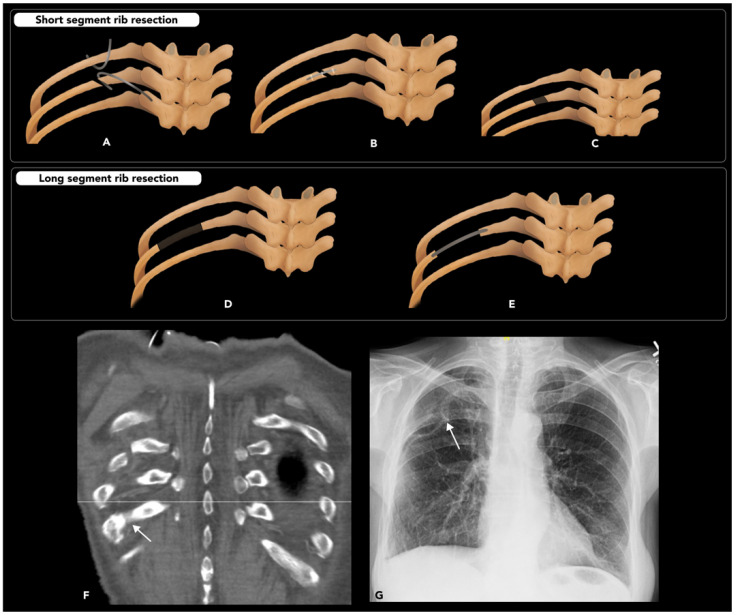
Rib osteotomy. (**A**–**D**) Short-segment rib resection: Illustrations showing the oblique osteotomy line with retractors in place (**A**), then rib fixation using hardware (**B**), and the final appearance post-resection (**C**). A coronal reconstructed CT image demonstrating the short-segment resection at the right 5th posterior rib (arrow (**F**)). (**D**–**E**) Long-segment rib resection: Illustrations showing the long-segment resection procedure and subsequent (**E**) wire fixation (grey, (**F**)). A posterior–anterior chest radiograph revealing the long-segment resection of the right 5th posterior rib (arrow (**G**)).

Rib osteotomy (RO) is performed as part of a posterolateral approach thoracotomy to access the contents of the thorax. This widely used technique, primarily by thoracic surgeons, creates a window to the lungs, hila, and mediastinum [97]. It may also occasionally be used to access the lumbar or lower thoracic spine [98]. Unlike other osteotomy techniques discussed prior in this review, RO is not performed as a primary corrective measure but rather to provide access for diagnostic or interventional procedures within the thorax. Care should be taken to remove only the minimum amount of rib necessary to ensure adequate exposure, thus reducing complications and promoting faster post-operative recovery. A unique aspect of the RO technique is that it involves dissecting the periosteum off the rib prior to performing the osteotomy. This maneuver helps mobilize the neurovascular bundle within the subcostal groove, protecting it from injury during the procedure. Once the periosteum is freed, a pair of cuts through the rib is then made to resect a segment of rib, often approximately 1 cm in length. At the end of the procedure, the defect in the rib may be bridged by a thin wire or plate and screw fixation, but the resected segment of rib is not replaced (Figure 17) [97].

Complications of RO may include damage to an intercostal nerve, which is a frequent cause of post-thoracotomy pain. Decreased mobility of the ribs and associated respiratory disturbances may also occur but can be minimized by removing a smaller segment of the rib. Other complications related to other steps of the thoracotomy or underlying illness may also be encountered [99].

## 3. Conclusions

In conclusion, it is essential for radiologists to have a thorough understanding of the various imaging findings associated with osteotomies across different imaging modalities for effective assessment, planning, and post-operative evaluation. Imaging techniques such as X-ray, CT scan, and MRI offer valuable insights into bone alignment, healing progression, and potential complications, all of which are crucial in guiding patient management and optimizing surgical outcomes.

## 4. Future Directions

Future research in pre- and post-operative imaging approaches for osteotomies should focus on integrating advanced imaging modalities with AI-based diagnostic tools and machine learning algorithms to identify at-risk patients, enabling early diagnosis of complications and timely intervention planning. Incorporation of dynamic imaging techniques could further provide real-time evaluation of the functional outcomes in the operated area, allowing for a more comprehensive assessment of joint stability and load distribution. Lastly, strengthening collaboration between radiologists and surgeons to implement structured reporting templates can standardize and unify interpretation during pre-operative evaluation and follow-up imaging. This approach enhances effective patient-centered care while minimizing the risk of over-treatment or sub-optimal treatment.

## Data Availability

No datasets were generated or analyzed during the current study.

## Abbreviations

The following abbreviations are used in this manuscript:

CORA	Center of rotation and angulation
ACA	Axis of correction of angulation
OWO	Opening-wedge osteotomy
CWO	Closing-wedge osteotomy
CT	Computed tomography
LNO	Lateral nasal osteotomy
LTL	Low-to-low
LTH	Low-to-high
RM	Reduction malarplasty
BSSO	Bilateral sagittal split osteotomy
IVRO	Intra-oral vertical ramus osteotomy
LO	Lingual osteotomy
BO	Buccal osteotomy
ERO	External rotation osteotomy
DISI	Dorsal segmental intercalated instability
OA	Osteoarthritis
HTO	High tibial osteotomy
ACL	Anterior cruciate ligament
PCL	Posterior cruciate ligament
TNO	Talar neck osteotomy
AVN	Avascular necrosis
CO	Calcaneal osteotomy
HV	Hallux valgus
MTP	Metatarsophalangeal
HVO	Hallux valgus osteotomy
HVA	Hallux valgus angle
IMA	Intermetatarsal angle
DDH	Developmental dysplasia of hip
LCA	Lateral center-edge angle
PAO	Peri-acetabular osteotomy
VO	Vertebral osteotomy
SVA	Sagittal vertical axis
PSO	Pedicle subtraction osteotomy
BDBO	Bone–disc–bone osteotomy
VCR	Vertebral column resection
RO	Rib osteotomy
